# N6-methyladenosine-related lncRNAs identified as potential biomarkers for predicting the overall survival of Asian gastric cancer patients

**DOI:** 10.1186/s12885-022-09801-z

**Published:** 2022-07-01

**Authors:** Shuyu Xu, Wenlong Chen, Yiwen Wang, Yuxin Zhang, Rong Xia, Jiemiao Shen, Xing Gong, Yinyin Liang, Jiayi Xu, Hua Tang, Tie Zhao, Yi Zhang, Tao Chen, Chao Wang

**Affiliations:** 1 Key Lab of Modern Toxicology of Ministry of Education, Center for Global Health, School of Public Health, Nanjing Medical University, 101 Longmian Avenue, Nanjing, 211166 People’s Republic of China; 2 State Key Lab of Reproductive Medicine, Institute of Toxicology, Nanjing Medical University, 101 Longmian Avenue, Nanjing, 211166 People’s Republic of China; 3 Department of Colorectal Surgery, the First Affiliated Hospital of Nanjing Medical University, Nanjing, 210000 People’s Republic of China; 4 The first clinical medical college of Nanjing Medical University, Nanjing, 211166 People’s Republic of China; 5 Department of General Surgery, Yueqing People’s Hospital, 338 Qingyuan road, Yueqing, 325600 Zhejiang province People’s Republic of China; 6 Department of General Surgery, Tongling People’s Hospital, 468 Bijiashan Road, Tongling, Anhui Province 244000 People’s Republic of China

**Keywords:** N6-methyladenosine (m6A), Long noncoding RNAs (lncRNAs), Gastric cancer, Prognostic signature, Asian patients

## Abstract

**Objective:**

Gastric cancer (GC) is one of the most prevalent malignant tumors in Asian countries. Studies have proposed that lncRNAs can be used as diagnostic and prognostic indicators of GC due to the high specificity of lncRNAs expression involvement in GC. Recently, N6-methyladenosine (m6A) has also emerged as an important modulator of the expression of lncRNAs in GC. This study aimed at establishing a novel m6A-related lncRNAs prognostic signature that can be used to construct accurate models for predicting the prognosis of GC in the Asian population.

**Methods:**

First, the levels of m6A modification and m6A methyltransferases expression in GC samples were determined using dot blot and western blot analyses. Next, we evaluated the lncRNAs expression profiles and the corresponding clinical data of 88 Asian GC patients retrieved from The Cancer Genome Atlas (TCGA) database. Differential expression of m6A-related lncRNAs between GC and normal tissues was investigated. The relationship between these target lncRNAs and potential immunotherapeutic signatures was also analyzed. Gene set enrichment analysis (GSEA) was performed to identify the malignancy-associated pathways. Univariate Cox regression, LASSO regression, and multivariate Cox regression analyses were performed to establish a novel prognostic m6A-related lncRNAs prognostic signature. Moreover, we constructed a predictive nomogram and determined the expression levels of nine m6A-related lncRNAs in 12 pairs of clinical samples.

**Results:**

We found that m6A methylation levels were significantly increased in GC tumor samples compared to adjacent normal tissues, and the increase was positively correlated with tumor stage. Patients were then divided into two clusters (cluster 1 and cluster 2) based on the differential expression of the m6A-related lncRNAs. Results showed that there was a significant difference in survival probability between the two clusters (*p* = 0.018). Notably, the low survival rate in cluster 2 may be associated with high expression of immune cells (resting memory CD4^+^ T cells, *p* = 0.027; regulatory T cells, *p* = 0.0018; monocytes, *p* = 0.00095; and resting dendritic cells, *p* = 0.015), and low expression of immune cells (resting NK cells, *p* = 0.033; and macrophages M1, *p* = 0.045). Enrichment analysis indicated that malignancy-associated biological processes were more common in the cluster 2 subgroup. Finally, the risk model comprising of six m6A-related lncRNAs was identified as an independent predictor of prognoses, which could divide patients into high- or low-risk groups. Time-dependent ROC analysis suggested that the risk score could accurately predict the prognosis of GC patients. Patients in the high-risk group had worse outcomes compared to patients in the low-risk group, and the risk score showed a positive correlation with immune cells (resting memory CD4^+^ T cells, R = 0.31, *P* = 0.038; regulatory T cells, R = 0.42, *P* = 0.0042; monocytes, R = 0.42, *P* = 0.0043). However, M1 macrophages (R = -0.37, *P* = 0.012) and resting NK cells (R = -0.31, *P* = 0.043) had a negative correlation with risk scores. Furthermore, analysis of clinical samples validated the weak positive correlation between the risk score and tumor stage.

**Conclusions:**

The risk model described here, based on the six m6A-related lncRNAs signature, and may predict the clinical prognoses and immunotherapeutic response in Asian GC patients.

**Supplementary Information:**

The online version contains supplementary material available at 10.1186/s12885-022-09801-z.

## Introduction

According to worldwide cancer statistics, gastric cancer (GC) is the fifth most common cancer and the third cause of cancer-related death in developing countries, with a very high incidence in Asian countries, especially in China [1]. A recent report indicated that the number of new GC cases reached one million in 2020, of which 769,000 patients succumbed [2]. Specifically, about 150–200,000 people die of GC in China every year according to the 2015 China Cancer Data Report [3]. Notably, there are different factors associated with the occurrence and progression of gastric cancer, including gene polymorphism, lifestyle such as drinking, smoking, high dietary salt intake, and iron deficiency [4], and *Helicobacter pylori* (*H. pylori*) infection, which is the strongest known risk factor for GC [5]. Evidence suggests that early detection and accurate non-invasive diagnosis of gastric cancer increases the chances of successful treatment [6], with a high 5-year overall survival (OS) rate (> 60%) in patients with early gastric cancer [7]. For these patients, surgical resection is the best treatment option. In contrast, patients with advanced stages of GC progression require chemotherapy, which is the most important treatment in metastasis [8]. Given the rapid technological advancement, immunotherapy and targeted therapy have been shown to improve the OS of GC patients [9]. Therefore, this calls for identification of the risk factors associated with GC patients’ outcomes, which will aid in classifying GC patients into different risk groups for individualized treatment, with the overarching goal of improving their treatment outcomes.

There are several biomarkers commonly used in GC, including carcinoembryonic antigen (CEA), alpha-fetoprotein (AFP), and carbohydrate antigen (CA) [10]. Considering the low sensitivity and positivity rate of the available biomarkers, many researchers have conducted studies to establish a new, stable, and powerful predictive model for early GC screening. Long non-coding RNAs (lncRNAs) in body fluids have attracted widespread attention as prognostic biomarkers due to their high specificity and sensitivity [11, 12]. Circulating lncRNAs have been associated with many prognosis-related factors (tumor size, stage, depth of invasion, and lymph node metastasis) and have been demonstrated as prognostic biomarkers of gastric cancer [13, 14]. Previous studies have revealed that several circulating exosomal lncRNAs, such as lncRNA *PCSK2–2:1* and *GNAQ-6:1*, are better biomarkers for distinguishing gastric cancer patients from healthy people compared to traditional diagnostic biomarkers, such as CEA and AFP [15, 16]. A recent study explored the landscape of lncRNAs associated with the immunome across 33 cancer types, and found that their expression level was correlated with the level of immune factors and immune cell infiltration [17]. This suggests that the development of an lncRNAs-based prediction model is not only beneficial for the prediction of OS in patients with early gastric cancer, but also for the selection of immunotherapy as the optimal treatment method for patients.

N6-methyladenosine (m6A), one of the most abundant internal modification in RNA, modulates several bioprocesses such as cell differentiation, DNA damage response, and carcinogenesis [18]. Studies have shown that m6A modification is important in the treatment of tumors, especially in targeted therapy [19]. The m6A modification is mediated by an m6A methyltransferase complex, composed of METTL3, METTL14, and WTAP, and is abolished by m6A demethylases, including ALKBH5 and FTO. At the same time, m6A-modification relies on reader proteins such as YTHDC1, YTHDC2, and YTHDF1 [20]. It is well known that, for tumor biology, the level of m6A modification changes significantly, and exerts its role in tumorigenesis, proliferation, metastasis, and immunoregulation, thereby influencing patients’ outcomes [21, 22]. Studies have reported that the level of m6A is significantly higher in hepatocellular carcinoma and gastric cancer patients than in normal individuals, and the level is positively correlated with the clinical outcome [23, 24]. However, in bladder cancer, a lower level of m6A is associated with a worse outcome [21]. This can be attributed to the fact that m6A modification has been identified on mRNA and some non-coding RNA, including mRNA, microRNA, lncRNA, snoRNA, and circRNA [25]. It is worth noting that m6A modification on RNA regulates the progress of oncogenesis and exerts critical roles in innate immunity [26], whereas m6A modification on lncRNA affects RNA-protein interaction [27]. For example, lncRNA *MALAT1*, associated with lung adenocarcinoma, was highly methylated with m6A [28]. Collectively, these studies suggest that m6A modification plays a critical role in regulating the expression of lncRNAs and may affect cancer development.

This study identified lncRNAs associated with m6A regulators, and analyzed their expression and related pathways in Asian gastric cancer patients. The six identified lncRNAs were then used to construct a prognosis model which could accurately predict the prognosis of patients, and may be potential biomarkers for classifying GC patients for targeted immunotherapy.

## Methods

### Clinical specimens

This study included GC patients admitted at the Tongling People’s Hospital from January 2018 to December 2019. Tissue specimens were collected from patients and immediately stored in liquid nitrogen, including 12 paired GC tumors and adjacent non-tumor tissues. Notably, signed informed consent was obtained from all study participants before enrollment. All participants had not received chemotherapy or radiotherapy before surgery. The study was approved by the Institutional Review Board of Nanjing Medical University and the Ethical committee of the Tongling People’s Hospital (ethical review No.2019–008) and was conducted in accordance with the Declaration of Helsinki.

### m6A dot blot analysis

Total RNA was separately extracted from human tissues using TRIzol (Invitrogen) reagent and the RNA concentration was determined using a NanoDrop 2000 (Thermo Fisher Scientific). The RNA concentration was then adjusted to 250, 500, and 1000 ng/μL. Two microliters of Poly(A) + RNAs were first denatured by heating at 65 °C for 5 min and spotted onto a nitrocellulose membrane (Amersham, GE Healthcare, USA). Next, the membrane was cross-linked by 1500 J UV, blocked with 5% non-fat milk twice, and incubated with m6A antibody (1:1000, Thermo Fisher Scientific, USA) overnight at 4 °C. Subsequently, the membrane was incubated with HRP-conjugated goat anti-mouse IgG dilution (1:3000, Proteintech, USA). Finally, the membrane was visualized using the chemiluminescence imaging analysis system (Tanon, Shanghai, China). The other membrane was dyed in 0.02% methylene blue (MB) for 30 min, followed by rinsing twice with ultrapure water and photographing. The membrane was visualized with chemiluminescence image analysis system (Tanon, Shanghai, China) and the grayscale was measured by ImageJ (NIH, Bethesda, MD) software.

### Western blot analysis

Western blotting experiments were performed in accordance with our previous study [29]. Briefly, tissues were harvested and lysed in RIPA buffer on ice, and the extracted protein was quantified by bicinchoninic acid (BCA) analysis (Beyotime, China). Next, proteins were resolved using 10% SDS-PAGE and transferred to polyvinylidene difluoride (PVDF) membranes (Millipore, USA). Nonspecific binding sites were then blocked by immersing the membranes in 5% bovine serum albumin in PBS at room temperature. Membranes were then incubated with the following high affinity primary antibodies: anti-WTAP antibody (1:1000, Cell Signaling Technology, USA), anti-METTL3 antibody (1:1000, Cell Signaling Technology, USA), anti-METTL14 antibody (1:1000, Cell Signaling Technology, USA), and anti-GAPDH antibody (1:1000, Cell Signaling Technology, USA). After washing, membranes were incubated with peroxidase (HRP)-conjugated secondary antibody (1:1000, Cell Signaling Technology, USA). Finally, protein signals were detected using a chemiluminescence imaging analysis system (Tanon, Shanghai, China).

### Acquisition of information on Asian GC patients

RNA sequence transcriptome data 88 Asian GC patients and their clinicopathological information were downloaded from the Cancer Genome Atlas (TCGA) data portal (https://cancergenome.nih.gov/). In addition, expression matrixes of 23 m6A-related genes were extracted from the TCGA database, including expression data on writers (*METTL3, METTL14, METTL16, WTAP, VIRMA, ZC3H13, RBM15,* and *RBM15B*), erasers (*FTO* and *ALKBH5*), and readers (*YTHDC1, YTHDC2, YTHDF1, YTHDF2, YTHDF3, HNRNPC, FMR1, LRPPRC, HNRNPA2B1, IGF2BP1, IGF2BP2, IGF2BP3,* and *RBMX*). The clinicopathological information for these samples is shown in Table 1 and supplementary Table S1.

**Table 1:** Clinical pathological parameters of Asian patients with gastric cancer in TCGA database

Clinical characteristic	All (*N* = 88)	N (%)
Age (years)	<=65	43 (48.9)
	> 65	45 (51.1)
Gender	Male	60 (68.2)
	Female	28 (31.8)
Stage	Stage I	8 (9.09)
	Stage II	41 (46.6)
	Stage III	34 (38.6)
	Stage IV	3 (3.41)
	Unknow	2 (2.27)
Grade	G1	4 (4.55)
	G2	30 (34.1)
	G3	53 (60.2)
	Unknow	1 (1.14)
T	T1	6 (6.82)
	T2	10 (11.4)
	T3	44 (50.0)
	T4	28 (31.8)
M	M0	84 (95.5)
	M1	3 (3.41)
	Unknow	1 (1.14)
N	N0	35 (39.8)
	N1	28 (31.8)
	N2	16 (18.2)
	N3	7 (7.95)
	Unknow	2 (2.27)
Vital Status	Alive	63 (71.6)
	Dead	25 (28.4)

### Prognostic signature construction

The long non-coding RNA annotation file of Genome Reference Consortium Human Build 38 (GRCh38) was acquired from the GENCODE website for annotation of the lncRNAs in the TCGA dataset. Pearson’s correlation analysis (with the |Pearson R| > 0.4 and *p* < 0.001) was used to screen m6A-related lncRNAs, which resulted in identification of 1054 m6A-related lncRNAs (supplementary Table S1). We used univariate Cox regression analysis combined with GC overall survival information in TCGA to explore the prognosis of m6A-related lncRNAs. In addition, limma and corrplot packages in R software were used to analyze the relationship between the differential expressions of lncRNAs, cancer-related factors, and immune factors in all samples. Gene Set Enrichment Analysis (GSEA) was then performed according to our previous study [30].

LncRNA expression profiles were normalized by [log2 (data + 1)]. Notably, the entire set included 72 patients since 16 patients with missing clinical data were excluded. The entire set was then randomly separated into the training set and the test set in a ratio of 5 to 5. Next, LASSO Cox regression and multivariate Cox proportional hazards regression models were performed to identify and analyze the prognostic genes in order to establish a predictive model**.** Notably, an m6A-related lncRNAs risk model was ultimately established. The following formula was used to calculate the risk score: Risk score = expression of lncRNA_1_ × β_1_lncRNA_1_ + expression of lncRNA_2_ × β_2_lncRNA_2_ + …expression of lncRNA_n_ × β_n_lncRNA_n_ (β represents the regression coefficient of each lncRNA). Two subgroups (low- and high- risk groups) were then established based on the median risk score. Subsequently, Kaplan-Meier curves and log-rank methods were used to evaluate the prognostic significance of the risk score. The receiver operating characteristic (ROC) curve analysis was used to evaluate the prognosis-prediction accuracy and risk score of each lncRNA, whereas the area under the curve (AUC) with cutoffs was used to measure prognosis prediction accuracy. Moreover, survival curves of clinic-pathologic characteristics and model validation between the two groups were generated using the Kaplan-Meier method. CIBERSORT algorithm was used to calculate the fractions of infiltrating immune cells using the ‘CIBERSORT’ R package (CIBERSORT R script v1.03). In addition, immune scores and stromal scores were obtained by the ESTIMATE algorithm using the ‘ESTIMATE’ R package (R package, v1.1.0). Tumor purity and infiltrating immune cells in Asian gastric cancer patients were analyzed in low- and high-risk groups. Finally, a model of the nomogram based on the final prediction model was constructed.

### Quantitative reverse-transcriptase PCR

Total RNA was extracted from tissues using TRIzol (Invitrogen) reagent. The integrity of isolated RNA was assessed by NanoDrop 2000 Spectrophotometer (Thermo Scientific, Wilmington, DE, USA). Next, RNA was reversely transcribed into cDNA using (Vazyme, Nanjing, China) according to the manufacturer’s protocol. RT-qPCR was then performed in the LightCycler96 (Roche) using SYBR Green technology. Relative lncRNA expression level was normalized to GAPDH and calculated using the 2^-∆∆Ct^ method. The primers for GAPDH and lncRNAs were purchased from Generay (Nanjing, China) and their sequences are shown in supplementary Table S2.

### Statistical analysis

All statistical analyses were performed using the R 3.6.3 and GraphPad Prism 7 software. Survival curves were plotted by Kaplan-Meier method, and differences in the survival rates between high-risk and low-risk groups were evaluated using the log-rank test. ROC curve and AUC were plotted by ‘SurvivalROC’ package in R. *P*-value < 0.05 was considered statistically significant.

## Results

### The level of m6A modification and expression of m6A regulators

**Fig. 1:**
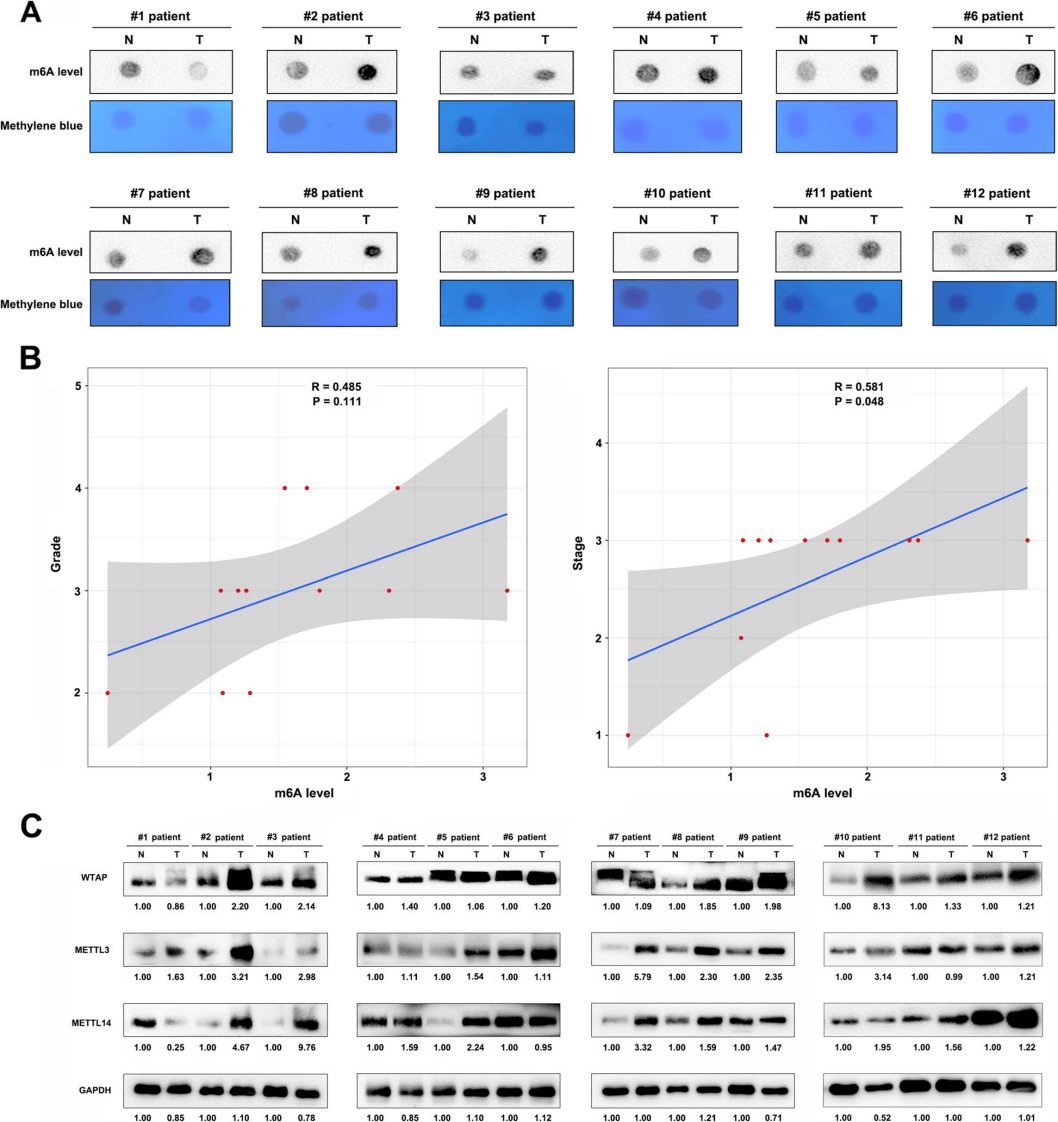
Expression profiles of m6A writer protein, its methylation levels in gastric cancer samples and correlation with clinical information. **A** Levels of m6A methylation in from gastric tumor samples alongside normal controls. **B** Correlation between levels of m6A methylation with grade, and tumor stages in gastric cancer samples. **C** Expression levels of METTL3, METTL14 and WTAP proteins in twelve paired gastric cancer tissue samples

We first evaluated the level of m6A modification in GC tissues using dot blot analysis. Results showed that expression of m6A level were higher in 11/12 patient carcinoma tissues compared to paracarcinoma tissues (Fig. 1 A). Then we quantified the m6A levels with densitometry from Fig. 1 A for correlation analysis. Correlation analysis also indicated that the tumor grade and clinical stage of patients were higher with increasing m6A level (R = 0.485, *P* = 0.111), especially in clinical stage (R = 0.581, *P* = 0.048) (Fig. 1 B). Given that m6A writers are important for cancer progression, usually as a tumor promoter during tumor progression, this study explored the expression of m6A writers, including METTL3, METTL14, and WTAP, in tumor and adjacent normal tissues. In 11/12 patient tumors, the expression of METTL3 and WTAP were higher compared to adjacent normal samples, and in 10/12 patient tumors, the expression of METTL14 were higher (Fig. 1 C). Overall, these results showed that the expression of METTL3, METTL14, and WTAP were higher in tumor samples than in the adjacent normal samples (supplementary Fig. S1).

### Identification of m6A-related lncRNAs in Asian GC patients

**Fig. 2:**
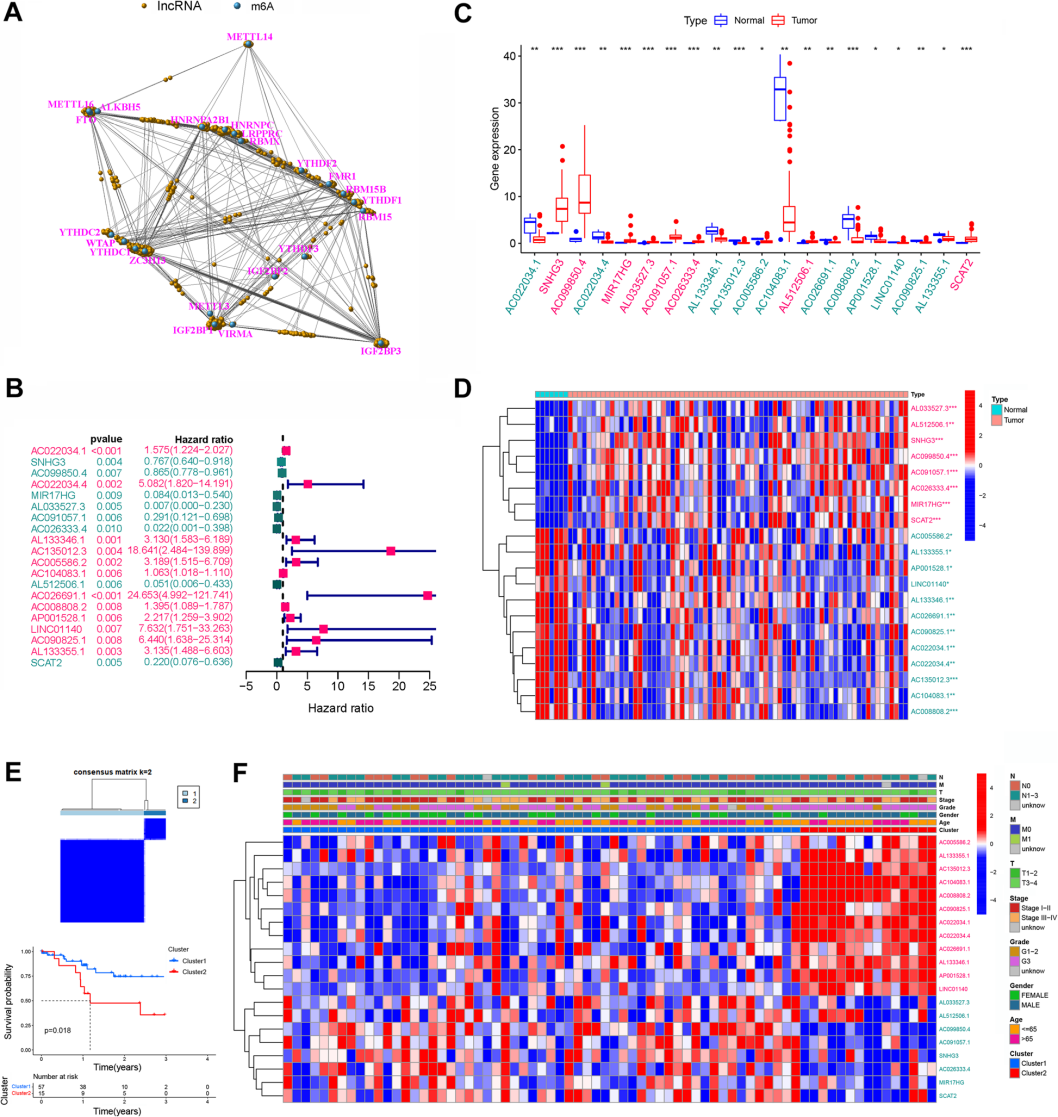
Screening and genotyping survival analysis of m6A-related lncRNAs related to the prognosis of gastric cancer. **A** A co-expression network of LncRNA-mRNAs. Blue and yellow nodes represent m6A-related mRNAs and lncRNAs, respectively. **B** Forest plot showing m6A-related lncRNAs significantly associated with prognosis of gastric cancer patients. Cyan and magenta fonts denote favorable and adverse prognostic factors, respectively. **C** Expression profiles of candidate lncRNAs in TCGA gastric cancer tissues. Red and blue boxes represent tumor and normal tissues, respectively, whereas cyan and magenta fonts denote downregulated and upregulated lncRNAs, respectively. **D** Heatmap showing differentially expressed lncRNAs between normal and tumor tissues. Magenta and cyan color show highly and lowly expressed lncRNAs, respectively. ^*^P < 0.05, ^**^P < 0.01, ^***^P < 0.001, relative to corresponding normal tissues. **E** Identification of two molecular subtypes based on the candidate lncRNAs and Kaplan-Meier survival curve analysis. **F** Heatmap and clinicopathologic features of the two clusters defined by the prognosis-related m6A-related lncRNAs expression

The matrix expression of 23 m6A genes and 1054 m6A-related lncRNAs was retrieved from the TCGA database (Fig. 2 A, supplementary Fig. S2 and supplementary Table S1). Univariate Cox regression analysis was the used to screen and identify the m6A-related lncRNAs associated with overall survival. The candidate lncRNAs were classified into two types: a risk type (*AC022034.1, AC022034.4, AL133346.1, AC135012.3, AC005586.2, AC104083.1, AC026691.1, AC008808.2, AP001528.1, LINC01140, AC090825.1, and AL133355.1*) with HR > 1, which was associated with poor prognosis, and a protective type (*SNHG3, AC099850.4, MIR17HG, AL033527.3, AC091057.1, AC026333.4, AL512506.1 and SCAT2*) with HR < 1, which was associated with good prognosis (Fig. 2 B, supplementary Table S1). Among the lncRNAs, eight were overexpressed in tumor tissues (*SNHG3, AC099850.4, MIR17HG, AL033527.3, AC091057.1, AC026333.4, AL512506.1, and SCAT2*) (Fig. 2 C and D). We clustered the 72 patients who were included in this study into two clusters using the ‘ConsensusClusterPlus’ package in R. When k = 2, two molecular subtypes were conducted (Fig. 2 E). The overall survival results showed that the patients’ prognosis was significantly different between the two subtypes (Fig. 2 E), with patients in cluster 2 exhibiting a significantly worse prognosis than those in cluster 1 (*P* = 0.018; Fig. 2 E). This suggested that the clinical results of the two subtypes were significantly different. However, the heatmap and clinic pathologic features showed that there were no significant differences in other clinical factors, including age, gender, grade, and TNM stage (Fig. 2 F).

### Distribution of tumor-infiltrating immune cells in Asian gastric cancer patients

**Fig. 3:**
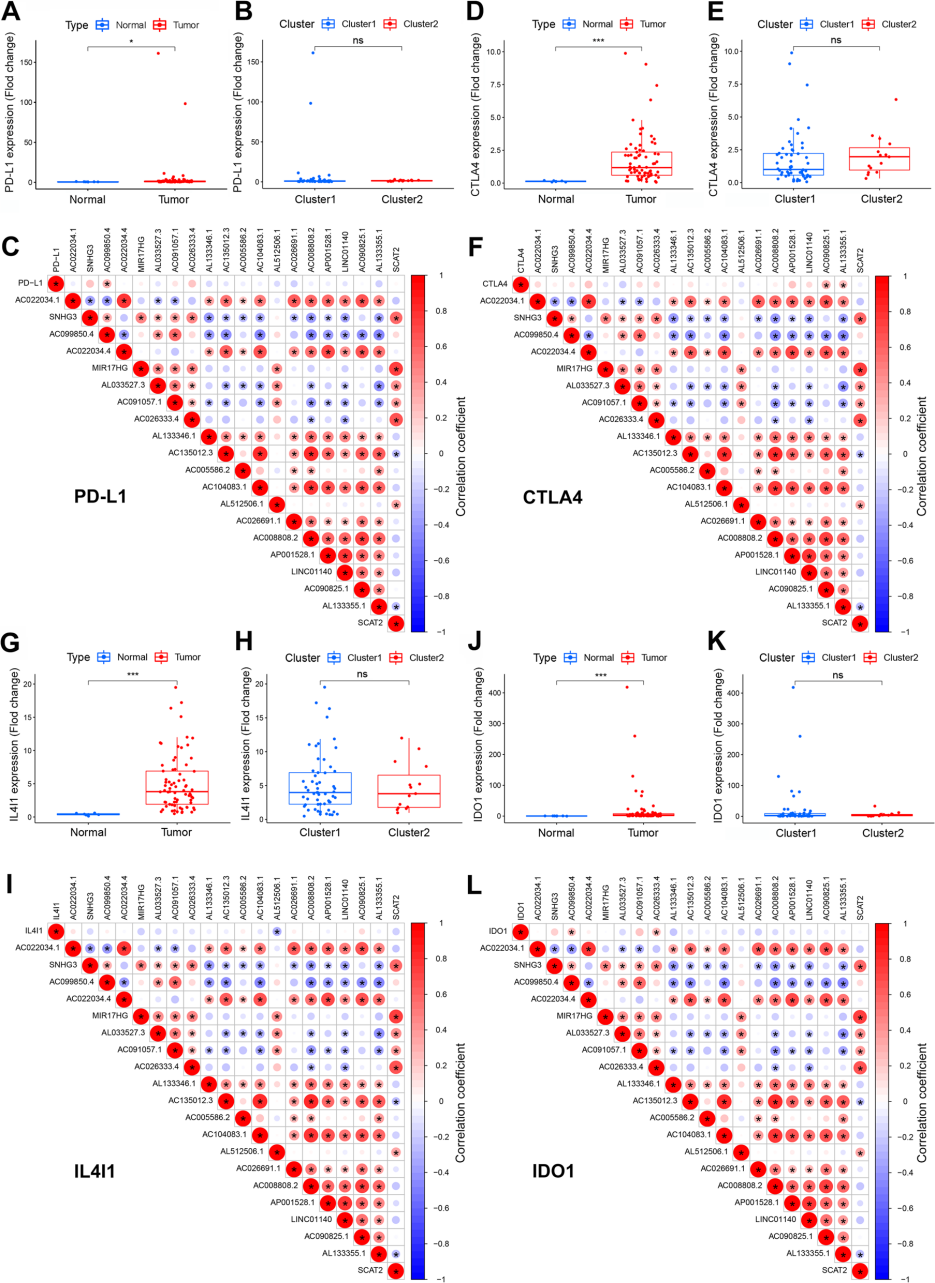
Expression profiles of four important immune checkpoints in gastric cancer tissues, and their correlation with prognosis-related m6A-related lncRNAs. Differences in the expression of PD-L1, CTLA4, IL4I1 and IDO1 in different tissue types (**A, D, G, J**) and various subtypes (**B, E, H, K**). Correlation analysis of the relationship between the expression of the target genes PD-L1, CTLA4, IL4I1 and IDO1 and that of prognosis-related m6A-related lncRNAs (**C, F, I, L**). Red indicates positive relationships, while blue indicates negative relationships. ^*^P < 0.05, ^***^P < 0.001

Next, we compared the expressions of four important immune checkpoints (PD-L1, CTLA4, IL4I1, and IDO1) between normal and tumor tissues, and between the two clusters. Results indicated that the four immune checkpoints exhibited increased expression in GC tumors. However, there was no significant difference between the two molecular subtypes (Fig. 3). Results also revealed a positive correlation between the expression of PD-L1 and *AC099850.4* (*P* < 0.05). There was a positive correlation between the expression of CTLA4 and *AC090825.1* and *AL133355.1* (*P* < 0.05), and a negative correlation between the expression of IL4I1 and *AL512506.1* (*P* < 0.05). Furthermore, the expression of IDO1 was significantly positively correlated with *AC099850.4* and *AC026333.4* (*P* < 0.05). Collectively, the results suggest that these lncRNAs were all tightly associated with tumor-immune microenvironment.

**Fig. 4:**
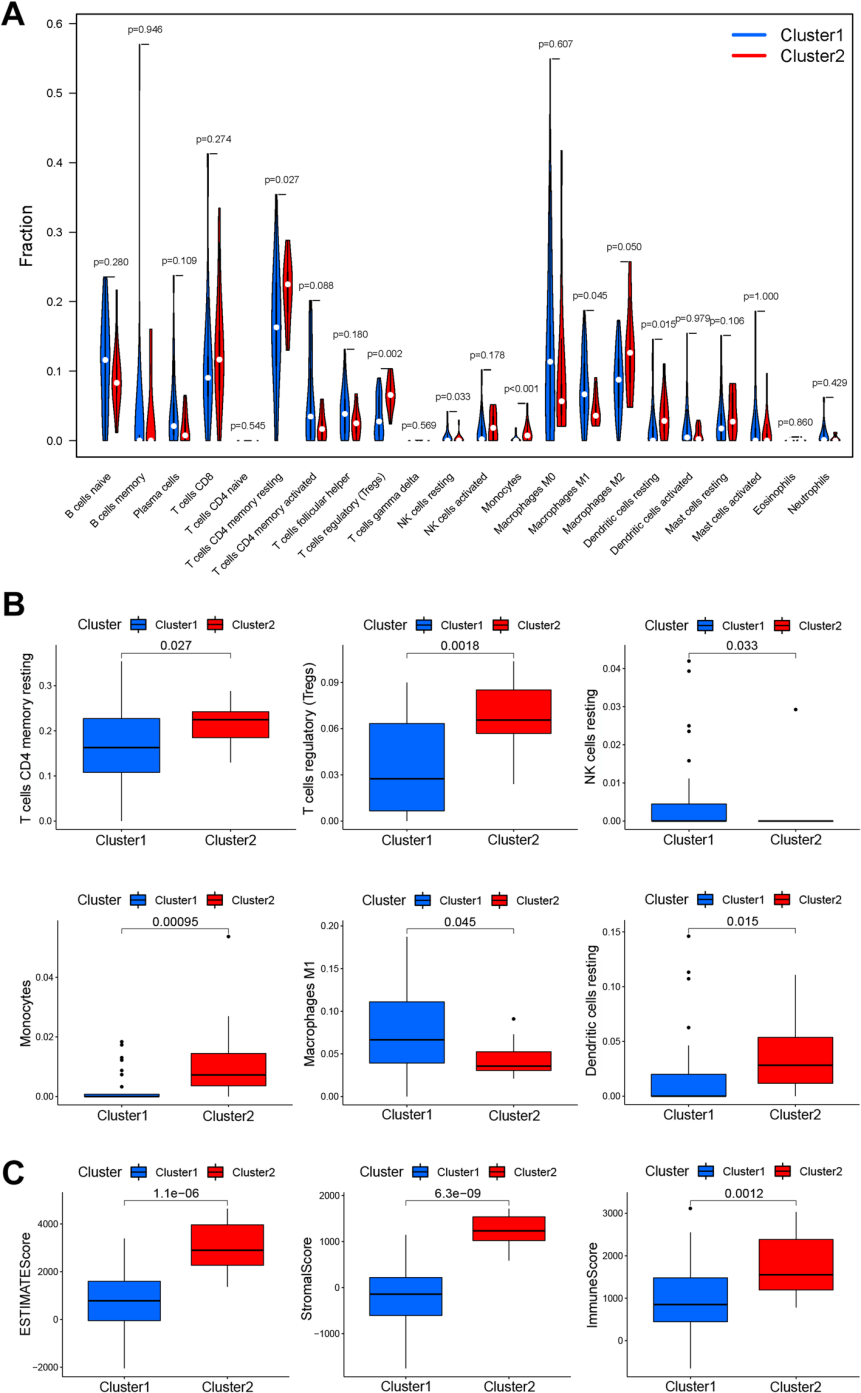
Proportions of 22 types of immune cells analyzed by the CIBERSORT algorithm, and estimate or stromal scores determined by the ESTIMATE algorithm. **A** Distribution and expression of 22 types of immune cells across the 2 molecular clusters. **B** Significantly differentially expressed immune cells in the two clusters. **C** ESTIMATE, stromal and immune scores in two clusters

In addition, the proportions of 22 types of immune cells were calculated using the CIBERSORT algorithm (Fig. 4 A). Patients in cluster 2 exhibited a worse prognosis, with increased immune cells (resting memory CD4^+^ T cells, *P* = 0.027; regulatory T cells, *P* = 0.0018; monocytes, *P* = 0.00095; and resting dendritic cells, *P* = 0.015), and decreased immune cells (resting NK cells, *P* = 0.033; and macrophages M1, *P* = 0.045) (Fig. 4 B). After ESTIMATE algorithm was processed, individuals in cluster 2 exhibited higher estimate score (*P* = 1.1e-6), higher stromal score (*P* = 6.3e-9), and higher immune score (*P* = 0.0012) than individuals in cluster 1 (Fig. 4 C).

**Fig. 5:**
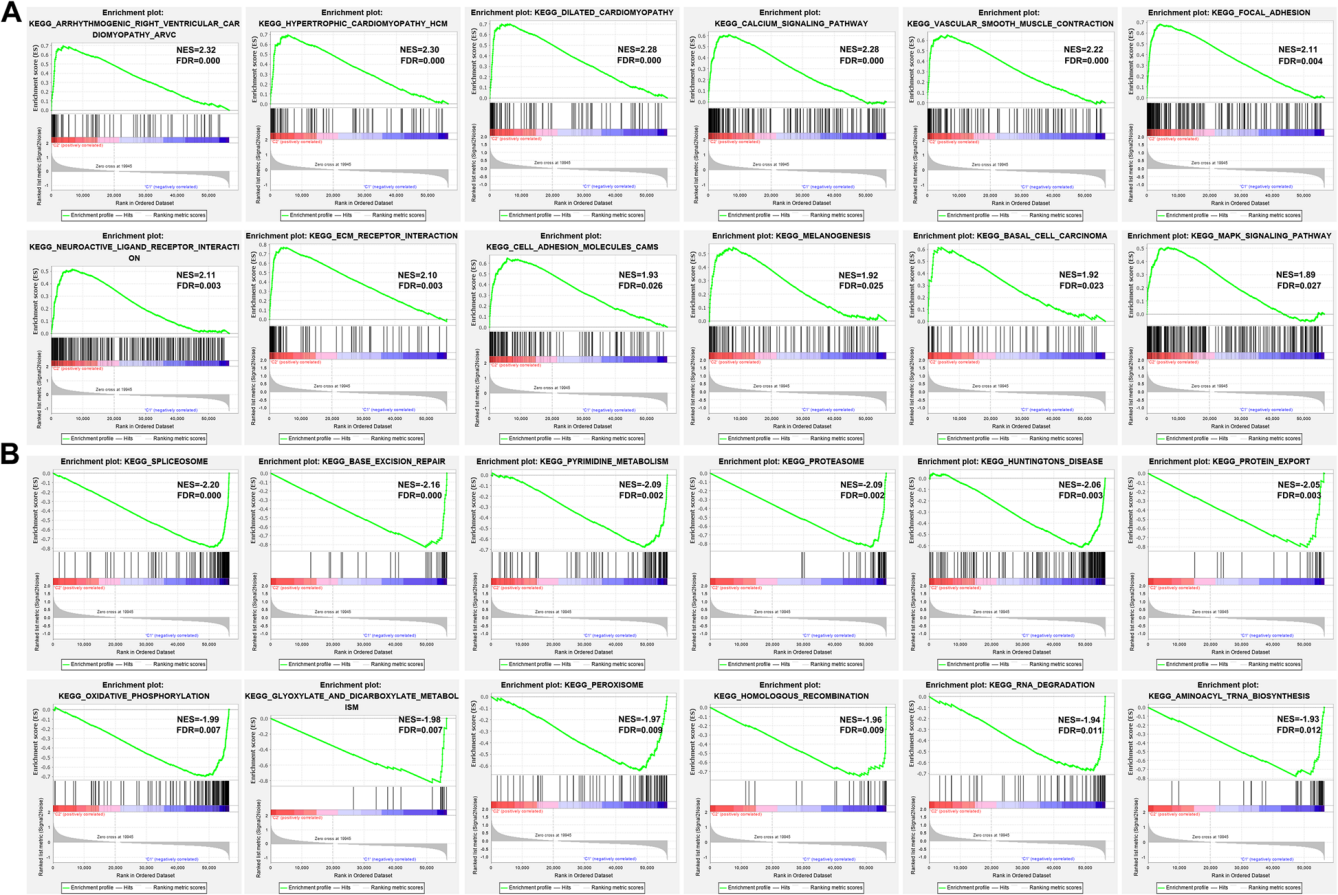
Gene set enrichment analysis (GSEA) results for Asian GC patients from TCGA datasets. **A** Tumor hallmarks were significantly enriched in the cluster 2 subgroup. **B** Significant enrichment of pathways related to cell cycle and cell damage repair in cluster 1 subgroup

Functional annotation was further performed through gene set enrichment analysis (GSEA). Results demonstrated that the genes involved in ECM_RECEPTOR_INTERACTION (NES = 2.10; FDR = 0.003), FOCAL_ADHESION (NES = 2.11; FDR = 0.004), CELL_ADHESION_MOLECULES_CAMS (NES = 1.93; FDR = 0.026) and MAPK_SIGNALING_PATHWAY (NES = 1.89; FDR = 0.027) were significantly enriched in cluster 2 individuals (Fig. 5 A). Meanwhile, the genes involved in BASE_EXCISION_REPAIR (NES = -2.15; FDR = 0.000), SPLICEOSOME (NES = -2.20; FDR = 0.000), HOMOLOGOUS_RECOMBINATION (NES = -1.96; FDR = 0.009), PROTEASOME (NES = -2.09; FDR = 0.002), and OXIDATIVE_PHOSPHORYLATION (NES = -1.99; FDR = 0.007) were significantly enriched in cluster 1 individuals (Fig. 5 B).

### Construction of the six m6A-related lncRNAs signature as an indicator for prognosis

**Fig. 6:**
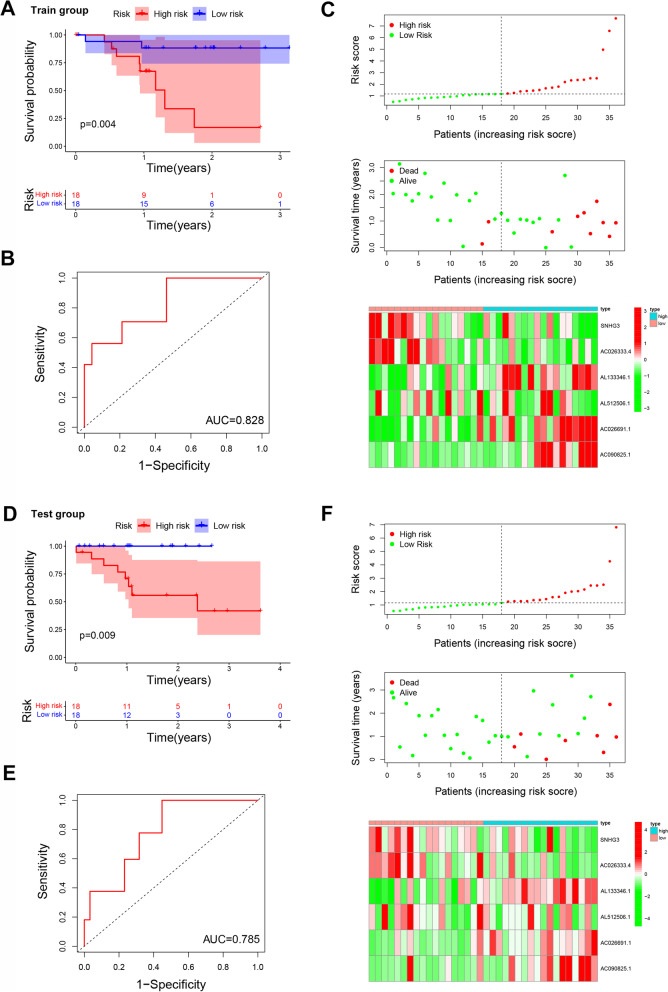
Construction and prognostic value of a nine m6A-related lncRNAs signature. **A** The overall survival rates of high- and low-risk groups in train group (*N* = 36). **B** Time-dependent ROC curves, based on risk scores, for predicting overall survival in the training dataset. **C** Distribution of risk scores, survival status and a heatmap of nine prognostic m6A-related lncRNAs in the training group. **D** Overall survival of patients in the high- and low-risk groups based on test dataset (*N* = 36). **E** Time-dependent ROC curves, based on risk scores, for predicting overall survival in the test group. **F **Distribution of risk scores, survival status and a heatmap of nine prognostic m6A-related lncRNAs in the test group

LASSO COX regression analysis was performed to optimize the prognostic model and prevent overfitting (supplementary Fig. S3). Finally, six m6A-related lncRNAs were screened as independent potential prognostic factors for OS and their risk coefficients are listed in Table 2. A prognostic model was constructed based on the expression of *SNHG3, AC026333.4, AL133346.1, AL512506.1, AC026691.1,* and *AC090825.1* (Table 2, supplementary Fig. S4). The risk score of gastric cancer prognosis was quantified using the following formula: risk score = *SNHG3*× (− 0.0203) + *AC026333.4*× (− 0.5629) + *AL133346.1*× (0.2539) + *AL512506.1*× (− 0.2812) + *AC026691.1*× (1.1868) + *AC090825.1*× (1.0361). Patients in the training set were then stratified into high-risk and low-risk groups based on the median risk score. Similarly, patients in the test set were also divided into low-risk and high-risk groups based on the cut-offs in the training set. Results showed that low-risk patients had longer OS than high-risk patients in both training set and test set (Fig. 6 A and D). ROC curve analysis showed that sensitivity was highest when AUC was 0.828 in the training set and 0.785 in the test set (Fig. 6 B and E). Moreover, the risk score and survival status predicted by the prognostic model were displayed in Fig. 6 C and F. From the results, it was evident that this model presented a good prediction power.

**Table 2:** Information on six prognostic m6A-related lncRNAs weighted by their multivariable LASSO regression coefficients

lncRNAs	Risk coefficient
*SNHG3*	−0.0203
*AC026333.4*	−0.5629
*AL133346.1*	0.2539
*AL512506.1*	−0.2812
*AC026691.1*	1.1868
*AC090825.1*	1.0361

### Relationships between the risk scores and clinical factors ​

**Fig. 7:**
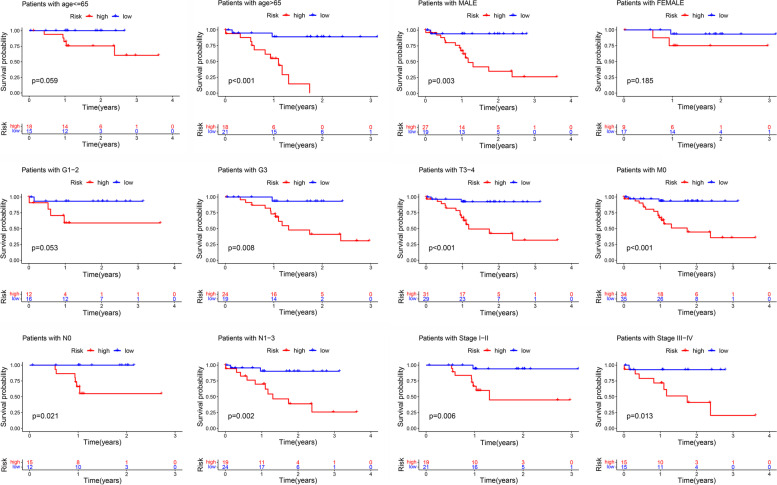
Kaplan-Meier survival curves for Asian GC patients in the TCGA dataset. Stratified analyses of clinicopathological factors in gastric cancer: age, gender, grade G1–2&G3, T3–4, M0, N0 & N1–3, and stage I-II & III-IV

**Fig. 8:**
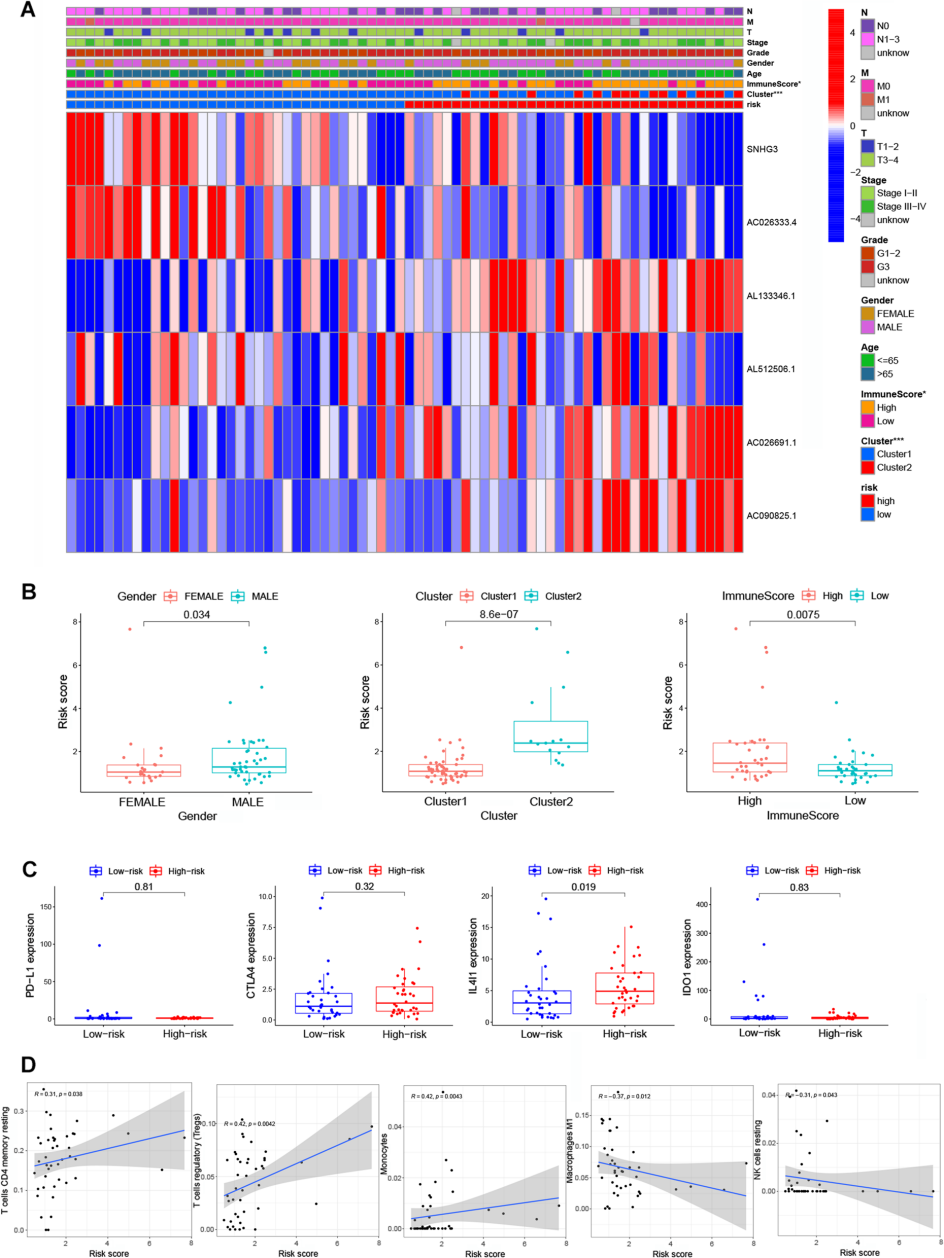
A heat map and box plot showing the relationship between risk scores with clinical features and immune cells. **A** A heatmap showing the correlation between risk scores and clinical characteristics. **B** Box plot showing the correlation between risk scores and clinical characteristics. **C** Profiles of PD-L1, CTLA4, IL4I1, and IDO1 expression in the high- and low-risk groups. **D** The correlation between risk scores with expression of immune cells, including resting memory CD4^+^ T cells, regulatory T cells, Monocytes, M1 macrophages and resting NK cells

Herein, we built a prognostic model based on the entire set to evaluate the risk score in different stratification. The model showed a good performance in the stratification of individuals aged less than 65 or more than 65, gender, grade G1–2&G3, T3–4, M0, N0&N1–3, and clinical stage I–II & III–IV (Fig. 7). These results showed a good performance in stratifying age ≤ 65 (*P* = 0.059) and > 65 (*P* < 0.001), male (*P* = 0.003) and female (*P* = 0.185), grade G1–2 (*P* = 0.053) and G3 (*P* = 0.008), T3–4 (*P* < 0.001), M0 (*P* < 0.001), N0 (*P* = 0.021) and N1–3 (*P* = 0.002), clinical-stage I-II (*P* = 0.006), and III-IV (*P* = 0.013) (Fig. 7). The high-risk group in both subgroups was associated with worse OS than the low-risk group in Asian gastric cancer patients, which was consistent with the above-described results. Figure 8 A shows the heatmap of the correlation analysis between risk scores and clinical traits. It was found that the gender, cluster, and immunescore had a significant positive correlation with the risk score. In addition, patients in the high-risk score group had higher immune scores (*P* = 0.0075), whereas all patients in cluster 2 had high-risk scores (*P* = 8.6e-07), which indicates worse prognostic results and suggests that patients in cluster 2 subgroup might benefit from immunotherapy. Meanwhile, results revealed that male patients had significantly higher risk scores than female patients (Fig. 8 B). Although, there was only difference in the expression of IL4I1 (*P* = 0.019), and no difference in the expression of other immune checkpoints (PD-L1, CTLA4 and IDO1) between the high- and low-risk score groups (Fig. 8 C), the results showed that the level of resting memory CD4^+^ T cells (*R* = 0.31, *P* = 0.038), regulatory T cells (Tregs) (*R* = 0.42, *P* = 0.0042), monocytes (*R* = 0.42, *P* = 0.0043) had a positive correlation with risk scores, whereas M1 macrophages (*R* = -0.37, *P* = 0.012) and resting NK cells (*R* = -0.31, *P* = 0.043) had a negative correlation with risk scores (Fig. 8 D).

### Construction of nomogram and its clinical utility

**Fig. 9:**
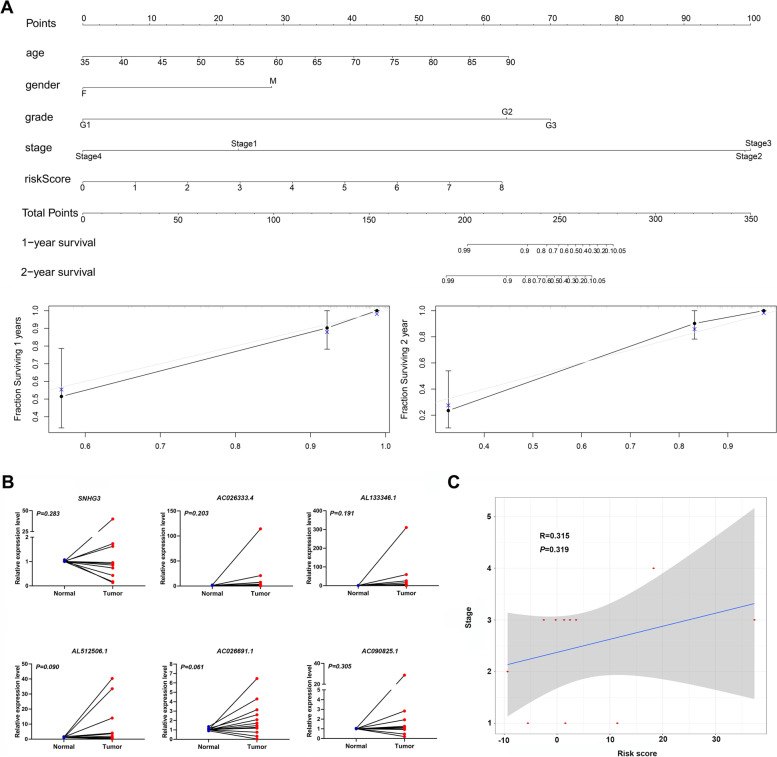
A novel nomogram for predicting overall survival rates of Asian GC patients based on independent prognostic factors and the nine m6A-related lncRNAs expression between normal and tumor tissues. **A** Nomogram for predicting overall survival probability of GC patients. **B** Expression profiles of six m6A-related lncRNAs in twelve paired gastric cancer tissue samples. C. Correlation between risk scores calculated by model with GC stage in patients

A nomogram was constructed to incorporate the risk score and clinical factors, and its accuracy was validated using the calibration curve of 1-year and 2-year survival (Fig. 9 A). Basic information and clinicopathological parameters of gastric cancer patients included in the study are shown in supplementary Table S3. qRT-PCR results validated the expression of the six predicted m6A-related lncRNAs (*SNHG3, AC026333.4, AL133346.1, AL512506.1, AC026691.1, and AC090825.1*) in clinical tissue samples (Fig. 9 B). Furthermore, a weak positive correlation was found between the risk score calculated by our model and stage of gastric cancer patients (*R* = 0.384, *P* = 0.218) (Fig. 9 C).

## Discussion

Currently, gastric cancer is a major gastrointestinal malignancy worldwide, with high incidence rates in East Asia [31]. It is worth noting that distinguishing the risk factors before treatment is important in improving patients’ outcomes. Therefore, this calls for identification of reliable biomarkers and establishing powerful prognostic models, with the overarching goal of improving the OS for GC patients. Although some models for predicting patient survival rates have been identified in recent years, they all have several limitations as described in our previous study [30]. Given the better stability and sensitivity of lncRNAs, they have become key biomarkers or drug treatment targets for GC patients [32, 33]. In many tumors, the lncRNAs signature is often used to predict the prognosis of tumor patients [34]. M6A methylation is the most prevalent post-transcriptional modification of lncRNAs, which affects RNA stability and translational activation [35]. Despite the elucidation of lncRNAs functions in gastric cancer, there is still less findings on the prognostic prediction model of lncRNAs’ m6A methylation modification.

Changes in the m6A modification have previously been implicated in the progression of oncogenesis, especially in gastric cancer [36]. Several studies have reported that progression of GC is promoted by m6A writers, for example, METTL3-mediated m6A modification of some mRNA (*HDGF* [24], *SPHK2* [37], and *MYC* [38]), which ultimately promotes gastric cancer progression, thus, exhibiting a prognostic significance. Meanwhile, many of the current studies are focusing on the role of m6A readers in lung cancer. One study found that m6A reader YTHDF1 facilitates the tumorigenesis and metastasis of GC via USP14 translation [39]. Knockdown of m6A reader IGF2BP3 inhibited hypoxia-induced cell migration and angiogenesis by regulating hypoxia inducible factor-1alpha in GC [40]. These findings suggest that abnormally high levels of m6A modification in GC patients are often positively correlated with poor prognosis, which is associated with high expression of writers and readers. This fact was also confirmed by our clinical tissue protein results and clinical correlation tests. M6A modification not only affects the functions of mRNAs, but also the lncRNAs. Notably, the functional link between lncRNA *GAS5* and m6A modification in colorectal cancer has already been proven, where the lncRNA *GAS5*, YAP signaling, and YTHDF3 formed a negative feedback loop that promoted cancer progression [41]. The m6A-mediated upregulation of *LINC01320* promotes the proliferation, migration, and invasion of gastric cancer via the miR495-5p/RAB19 axis [42]. However, although recent studies have paid more attention on the correlation between m6A modification and lncRNAs, further research is still needed.

Herein, it was found that higher level of m6A modification and selected lncRNAs in human gastric tumor were associated with poor OS. Notably, we screened out six lncRNAs, including *SNHG3, AC026333.4, AL133346.1, AL512506.1, AC026691.1, and AC090825.1*. LncRNA *SNHG3* is a oncogenic and an abnormally expressed lncRNA in various tumors such as gastric cancer [43] and lung cancer [44]. Aberrant expression of *SNHG3* is correlated with worse outcomes of patients through contributing to tumor cell proliferation, migration, and invasion [45]. A previous study proposed that *SNHG3* could be used as an independent prognostic biomarker of intrahepatic cholangiocarcinoma [46]. AL133346.1 and neighboring protein CCN2 were high-expressed in leukemia samples and could be used to predict the prognosis of patients with B-cell acute lymphoblastic leukemia [47]. AL512506.1 had been found as m6A-related lncRNA and used to establish risk assessment model with other lncRNAs in gastric cancer patients [48]. These findings are in line with our results, which provides further evidence on the potential functions of those lncRNAs and their potential capacities as biomarkers in cancer treatment. However, to date, studies about their molecular mechanism in Asian gastric cancer patients are rare.

After analyzing the relationship between lncRNAs and immune checkpoints, it was found that lncRNAs were closely associated with immune scores. Several immune factors, including PD-L1, CTLA4, IL4I1, and IDO1, were significantly enriched in GC tumors and were correlated with the six identified lncRNAs. The enrichment of CTLA4 and IL4I1 were consistent with their functions in cancer [49]. For example, programmed cell death 1 (PD-L1), cytotoxic T lymphocyte antigen 4 (CTLA4), and the indoleamine 2, 3-dioxygenase 1 (IDO1) have achieved impressive success in the treatment of different cancer types, especially the combined treatment of PD-L1 and CTLA4 [49]. In a previous study, CTLA4, PD-1, and PD-L1, as the checkpoint molecules, could be targeted by antibodies to slow cancer progression [50]. CTLA4 could affect diversity of T cells through increased T cell priming, and acted on both cytotoxic T cells and helper T cells [51]. In gastric cancer, significant benefits were observed after inhibiting PD-L1 and CTLA4 [52]. On the other side, the expression of interleukin-4induced-1 (IL4I1) in tumors has also been observed very frequently, especially in tumor-associated macrophages [53]. IL4I1 can activate aryl hydrocarbon receptor (AHR) and promote progression of cancer by enhancing chronic lymphocytic leukemia progression [54]. Similarly, IDO1 can promote AHR-driven processes and is associated with worse outcomes for tumor patients [55]. However, the function of these genes in cancer is still controversial, and thus more studies should be conducted to clarify their functions. Meanwhile, the poorer prognosis may be associated with the abnormally high expression of three immune cells (resting memory CD4^+^ T cells, regulatory T cells and monocytes) and down expression of two immune cells (M1 macrophages and resting NK cells), especially in Asian males with gastric cancer. Activated regulatory T cells accumulated in tumor microenvironment and correlated with tumor progression in patients with colorectal cancer [56]. Monocytes and macrophages are the major sources of numerous cytokines, including tumor necrosis factor alpha (TNF), interleukins, colony-stimulating factors, chemokines and cytotoxic mediators, which appear to play an important role in the regulation of tumor growth [57]. Notably, tumor-infiltrating NK cells are mostly “resting” non-activated and a component of innate immunity [58]. Studies report that reduced NK cell activity in gastric cancer patients is associated with poorer prognosis [59]. Collectively, the findings of this study suggest that our prognosis prediction model can identify Asian gastric cancer patients who may benefit from immunotherapy, thereby prolonging their overall survival. Further validation of the six m6A-related lncRNAs signature in a large sample population is necessary and more data is be needed to validate the general applicability of this signature in clinical decisions.

## Conclusions

This study has combined clinical characteristics with the expression of six m6A-related lncRNAs, including *SNHG3, AC026333.4, AL133346.1, AL512506.1, AC026691.1, and AC090825.1*, to construct an accurate prognosis prediction model for predicting the overall survival of Asian GC patients.

## Supplementary Information

**Additional file 1:** **Supplementary Figure S1-S4.**

**Additional file 2:** **Supplementary Table S1.**

**Additional file 3:** **Supplementary Table S2.**

**Additional file 4:** **Supplementary Table S3.**

## Data Availability

The data sets used and/or analyzed during the current study are publicly available data from The Cancer Genome Atlas (TCGA, http://cancergenome.nih.gov/). The figures and materials supporting the conclusions of this article are included within the article.

## Abbreviations

GC: Gastric cancer
TCGA: The cancer genome atlas
m6A: N6-methyladenosine
GSEA: Gene set enrichment analysis
LncRNAs: Long noncoding RNAs
CEA: Carcinoembryonic antigen
AFP: Alpha-fetoprotein
CA: Carbohydrate antigen
ROC: The receiver operating characteristic curve
AUC: The area under the curve
OS: Overall survival
PD-L1: Programmed cell death 1
CTLA4: Cytotoxic T lymphocyte antigen 4
IDO1: Indoleamine 2, 3-dioxygenase 1
IL4I1: Interleukin-4 induced-1
