# Supplementary material for: N6-methyladenosine-related lncRNAs identified as potential biomarkers for predicting the overall survival of Asian gastric cancer patients
Source: BMC Cancer. 2022 Jul 1;22:721. doi: 10.1186/s12885-022-09801-z (PMC9248105; doi:10.1186/s12885-022-09801-z)

**Supplementary information**

**Figure S1.** The quantification of the protein bands were performed using densitometric scanning analysis. **A.** METTL3. **B.** METTL14. **C.** WTAP.


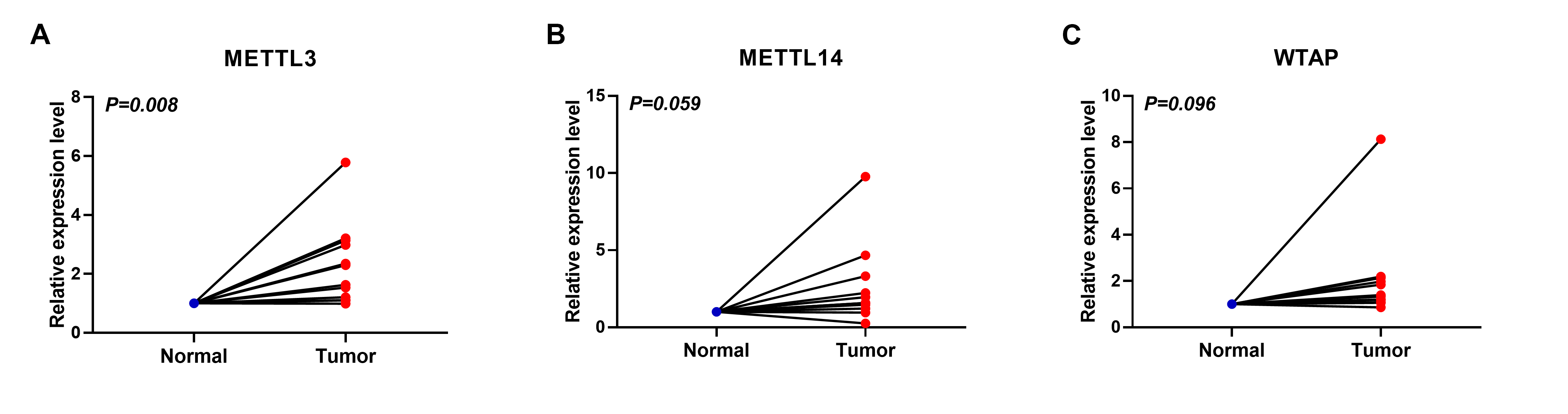


**Figure S2.** Sankey diagram showing the correlation between 23 m6A genes with 1054 m6A-related lncRNAs, as abstracted from the TCGA database.


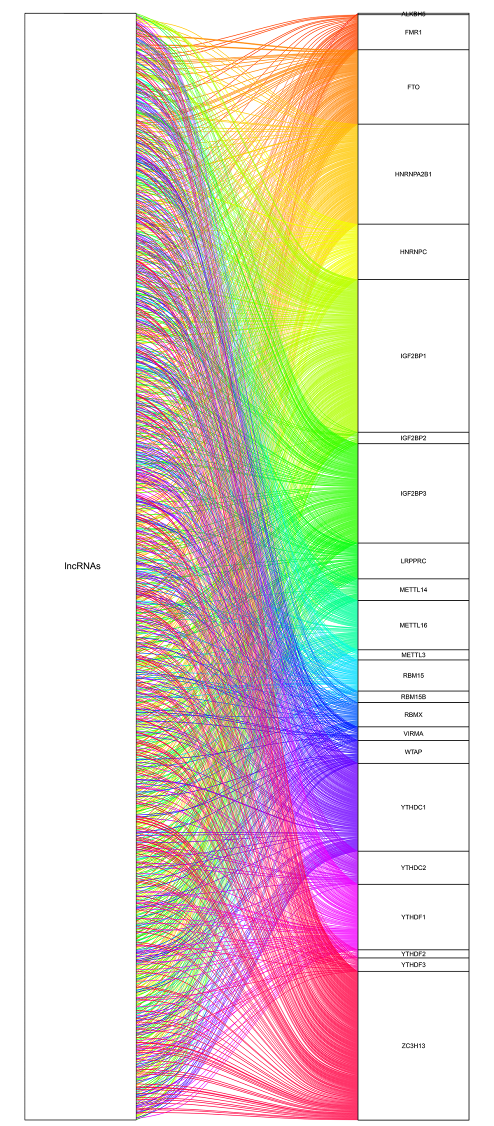


**Figure S3.** A prediction model established based on LASSO Cox regression analysis. **A.** Curves represent regularization paths of LASSO coefficients. **B.** Partial likelihood deviance as a function of regularization parameter λ in the TCGA dataset.


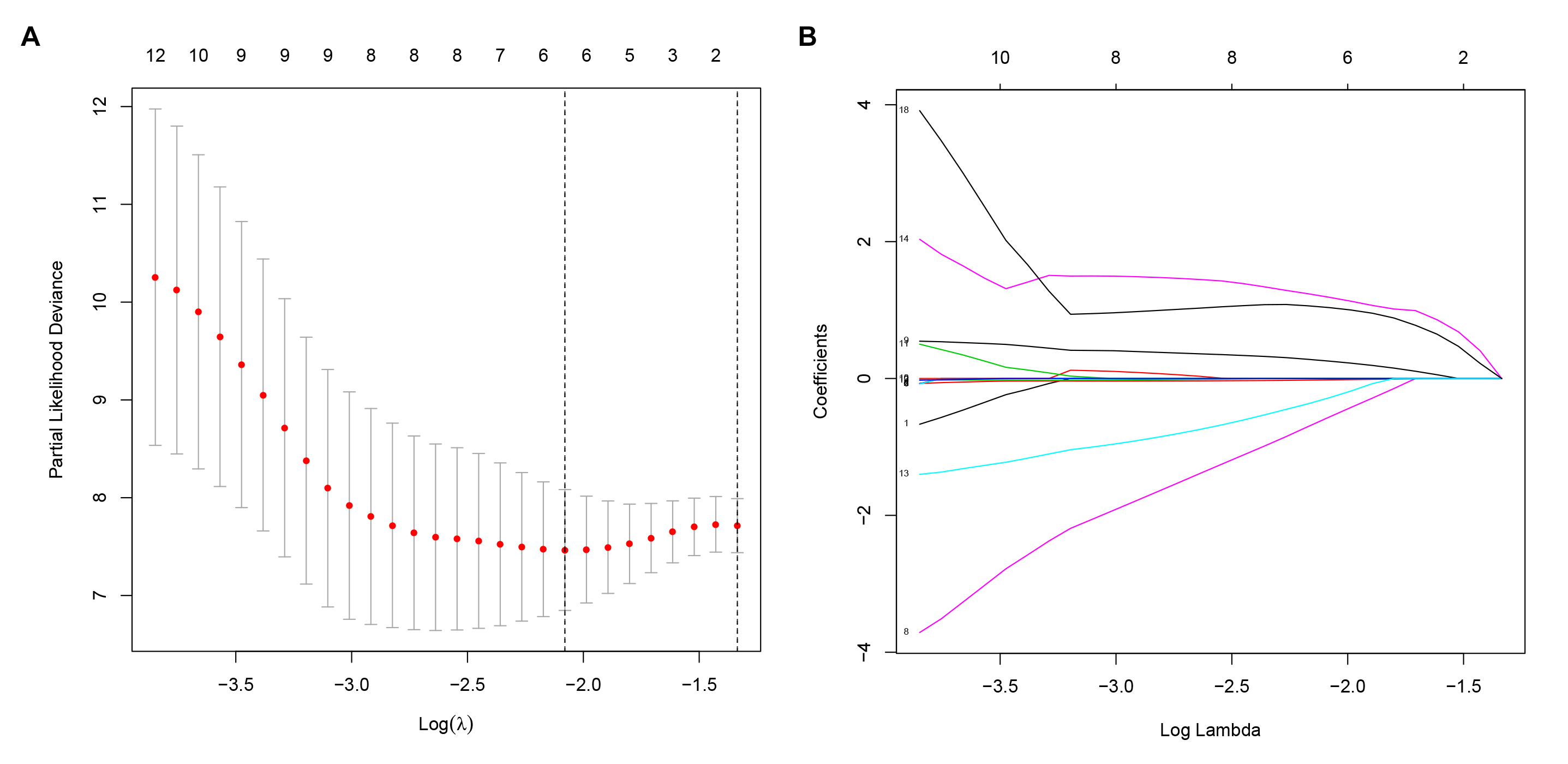


**Figure S4.** Sankey diagram showing the relationship among six m6A-related lncRNAs screened as independent potential prognostic factors for OS.


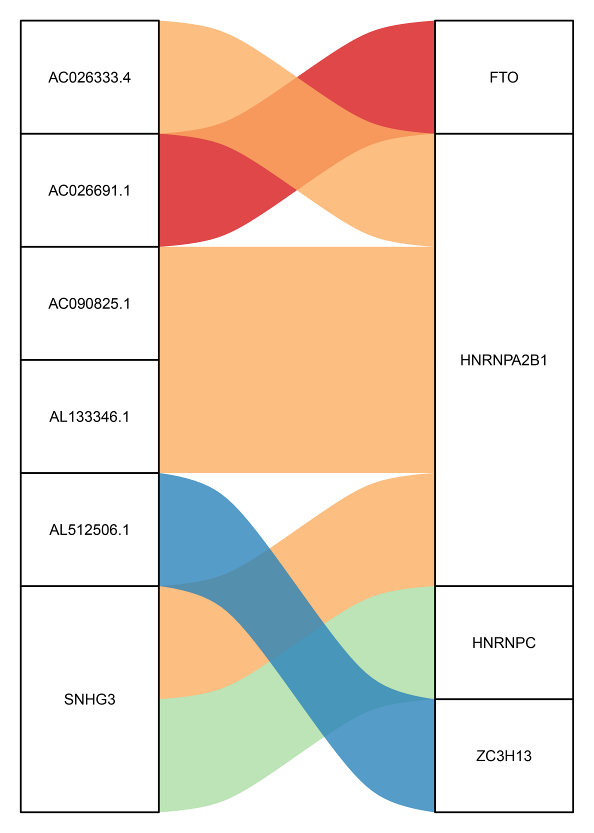

Supplement: Supplementary file 1 — Additional file 1: Supplementary Figure S1-S4. [file 12885_2022_9801_MOESM1_ESM.docx]
