# Supplementary material for: N6-methyladenosine-related lncRNAs identified as potential biomarkers for predicting the overall survival of Asian gastric cancer patients
Source: BMC Cancer. 2022 Jul 1;22:721. doi: 10.1186/s12885-022-09801-z (PMC9248105; doi:10.1186/s12885-022-09801-z)
Supplement: Supplementary file 3 — Additional file 3: Supplementary Table S2. [file 12885_2022_9801_MOESM3_ESM.docx]

**Supplementary Table S2. The information of primer sequence used in qRT-PCR.**

| LncRNAs | Forward 5′→3′ primer | Reverse 5′→3′ primer |
| --- | --- | --- |
| SNHG3 | **ACTTCCGGGCACTTCGTAAG** | **TGAGTGACAAACCGACTCCG** |
| AC026333.4 | **TATAGTGCTGCCTTGTAG** | **TTCTTAGTGGAGTTGGTC** |
| AC026691.1 | **GGGACTTCACTAAGCACCCC** | **CACTGCAGTGCTTGGTTCAC** |
| AC090825.1 | **TCTCTCTGGGAGACTGAGGC** | **CCCTCCCAGGTTCAAGCAAT** |
| AL133346.1 | **ACCTGTGCCTGCCATTACAACTG** | **CATGCCATGTCTCCGTACATCTTCC** |
| AL512506.1 | **AGATAGAGCTGGACGGGTGT** | **CCTCCAGCCTGCTCTTTCAT** |
| GAPDH | **GAAGGTGAAGGTCGGAGTC** | **GAAGATGGTGATGGGATTTC** |
