# Supplementary material for: N6-methyladenosine-related lncRNAs identified as potential biomarkers for predicting the overall survival of Asian gastric cancer patients
Source: BMC Cancer. 2022 Jul 1;22:721. doi: 10.1186/s12885-022-09801-z (PMC9248105; doi:10.1186/s12885-022-09801-z)
Supplement: Supplementary file 4 — Additional file 4: Supplementary Table S3. [file 12885_2022_9801_MOESM4_ESM.docx]

**Supplementary Table S3. Clinical pathological parameters of patients with gastric cancer.**

| **Number** | **Age** | **Gender** | **Grade** | **Stage** | **T** | **M** | **N** |
| --- | --- | --- | --- | --- | --- | --- | --- |
| **1** | 63 | Male | G2 | 1 | T1 | M0 | N1 |
| **2** | 49 | Male | G4 | 3 | T4a | M0 | N1 |
| **3** | 70 | Male | G2 | 1 | T2 | M0 | N0 |
| **4** | 74 | Male | G2 | 3 | T4a | M0 | N0 |
| **5** | 63 | Male | G3 | 2 | T3 | M0 | N0 |
| **6** | 69 | Male | G3 | 3 | T4a | M0 | N1 |
| **7** | 73 | Male | G3 | 1 | T1 | M0 | N0 |
| **8** | 84 | Female | G3 | 3 | T4b | M0 | N1 |
| **9** | 62 | Male | G3 | 3 | T3 | M0 | N2 |
| **10** | 40 | Male | G4 | 3 | T4a | M0 | N2 |
| **11** | 45 | Female | G2 | 3 | T4 | M0 | N1 |
| **12** | 83 | Male | G4 | 3 | T4b | M0 | N1 |
